# Early anastomotic complications in colorectal surgery: a systematic review of techniques for endoscopic salvage

**DOI:** 10.1007/s00464-019-06670-9

**Published:** 2019-01-23

**Authors:** R. E. Clifford, H. Fowler, N. Govindarajah, D. Vimalachandran, P. A. Sutton

**Affiliations:** 1 Institute of Cancer Medicine, University of Liverpool, Liverpool, L69 3GE UK; 2 The Countess of Chester Hospital NHS Foundation Trust, Chester, UK

**Keywords:** Colorectal, Anastomotic leak, Stricture, Colonoscopy, Endoscopy

## Abstract

**Background:**

Anastomotic complications following colorectal surgery are associated with significant morbidity and mortality. For patients in whom systemic sepsis is absent or well controlled, minimal access techniques, such as endoscopic therapies, are being increasingly employed to reduce the morbidity of surgical re-intervention. In this review, we aim to assess the utility of endoscopic management in the acute setting of colorectal anastomotic complications, focusing on anastomotic leak.

**Method:**

A literature search was performed for published full text articles using the PubMed, Cochrane and Scopus databases using the search criteria string “colorectal anastomotic (“leak” OR “bleed”), “endoscopy”, endoscopic management”. Additional papers were detected by scanning the references of relevant papers. Data were extracted from each study by two authors onto a dedicated pro-forma. Given the nature of the data extracted, no meta-analysis was performed.

**Results:**

A total of 89 papers were identified, 16 of which were included in this review; an additional 14 papers were obtained from reference searches. In patients who are not physiologically compromised, there are promising data regarding the salvage rate of stents, over-the-scope endoscopic clips, vacuum therapy and fibrin glue in the early management of colorectal anastomotic leak. There is no consensus regarding the optimal approach, and data to assist the physician in patient selection are lacking. Whilst data on salvage (i.e. healing and avoidance of surgery) are well understood, no data on functional outcomes are reported.

**Conclusion:**

Endoscopic therapy in the management of stable patients with colorectal anastomotic leaks appears safe and in selected patients is associated with high rates of technical success. Challenges remain in selecting the most appropriate strategy, patient selection, and understanding the functional and long-term sequelae of this approach. Further evidence from large prospective cohort studies are needed to further evaluate the role of these novel strategies.

There are 41,000 patients diagnosed with colorectal cancer in the UK each year [1]. Many of these patients will undergo surgical resection with the formation of an anastomosis, often at the greatest risk below the pelvic brim for rectal cancer resection. Despite advances in surgical technique, anastomotic complications continue to be associated with a significant rate of morbidity and mortality; including potential permanent stoma formation, increased length of hospital stay [2], increased local recurrence [3] and significant financial implications for an ever strained health service. Anastomotic leaks occur in 5–15% of patients following a colorectal anastomosis [4–6] and are more frequently observed in those of a male sex, a BMI > 35 kg/m^2^, those who have had pre-operative chemo-radiation, or patients with tumours > 5 cm in size or within 7 cm of the anal verge [7]. Intraoperative assessment of anastomotic integrity is now common practice, whether by an air leak test, endoscopy, intraoperative dye test or laser fluorescence angiography. However, techniques to then subsequently reduce the leak rate have little evidence, including transanal decompression devices, intraluminal barriers or extraluminal devices such as tissue bolstering. The use of drains and mechanical bowel preparation also continues to be a subject of debate [8, 9].

In patients who are physiologically unwell, the traditional treatment for the disruption of a colorectal anastomosis is to return to theatre for lavage and take down of the anastomosis; however, this has increasingly been the subject of some debate [10, 11]. Whilst this removes the source of sepsis, patients undergo a second major operation carrying a morbidity in excess of 50% [11]. The “divert and drain” approach of a defunctioning loop ileostomy and pelvic drainage, whilst leaving the anastomosis intact, has proved increasingly popular with a success rate ranging upwards from 54% [12]. Leaving the primary anastomosis intact avoids more complex dissection in inflamed tissue planes and has been shown to be associated with a threefold increase in the likelihood of patients achieving stoma reversal [13]. Conversely, salvaging an anastomosis in this manner may predispose patients to chronic pelvic sepsis and poor functional outcomes [14, 15].

There has been increasing interest in methods of anastomotic salvage which do not require re-entry into the abdominal cavity. In selected patients, endoscopy may provide the advantage of a diagnostic element with several options for safe therapeutic management without precluding second line invasive surgical options. In this review, we aim to assess the utility of endoscopic management in the acute setting of colorectal anastomotic complications, focusing on anastomotic leak.

## Methods

A literature search was performed for published full text articles using the PubMed, Cochrane and Scopus databases using the search criteria string “colorectal anastomotic (“leak” OR “bleed”), “endoscopy”, endoscopic management”. Additional papers were detected by scanning the references of relevant papers. Search results were initially included due to a relevant title, and those papers were then read through in full. All study types were included although the search was limited to papers with a focus on colorectal surgery. Exclusion criteria included those reporting only on anastomoses of the upper gastro-intestinal tract. Papers were reviewed using the Covidence™ system (http://www.covidence.org) to enable reviews to take place methodically.

Once eligible papers were identified, a search was performed to exclude duplicated results or duplicated data sets to produce the final list of papers included. Data were extracted from each study by two authors onto a dedicated pro-forma. Given the heterogenous nature of the data extracted, no meta-analysis was performed. As secondary research, no institutional approval was required within the United Kingdom.

## Results

**Fig. 1:**
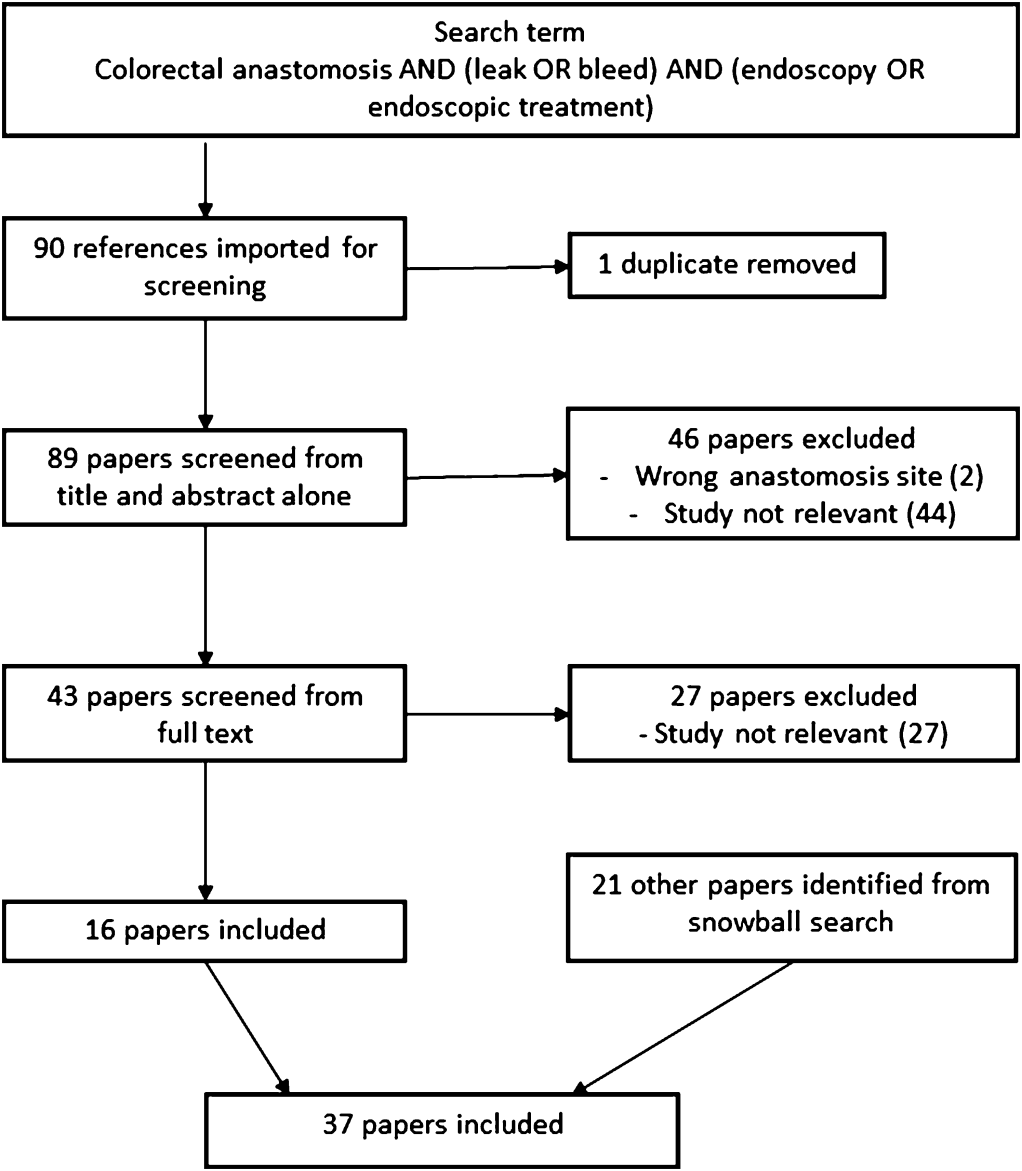
PRISMA diagram

A total of 89 papers were identified, 16 of which were included in this review; additional 21 papers were obtained from reference searches. These include 3 systematic reviews, 4 cohort studies, 28 case series, 1 case report and 1 pilot study. There were no randomised trials. Figure 1 shows the PRISMA flow diagram. Nine papers included patients with anastomotic leaks managed using stents, seven endoscopic clips, 14 using vacuum therapy, three fibrin glue and four on multi-modal management of anastomotic bleeding. Other papers reviewing a combination of therapies have been referenced throughout.

### Stenting

Self-expanding metal stents (SEMS) have been considered for the use of colorectal surgical complications for many years. Stents can vary in terms of their silicon coverage (fully, partially, uncovered) and material (metal or biodegradable). The aim with anastomotic leak is to place the stent across the defect to prevent communication between the lumen and extraluminal space to protect the patient from sepsis during tissue growth [16].

There were a total of nine case series or cohort studies including 58 patients who had their anastomotic leaks managed with stents (Table 1) [5, 17–24]. Long-term salvage rates were reported between 50 and 100%, which in most cases was defined as evidence of closure at time of removal of the stent or follow-up endoscopy. The largest cohort study to date focusing on the use of stents for anastomotic leak was by Lamazza et al. [22] This study included 22 patients with an anastomotic defect greater than 30% of the circumference, which was confirmed on a low-pressure gastrografin enema study. 68% of their patients had a defunctioning stoma after the diagnosis of leak and 27% of patients had to undergo repeat stenting after spontaneous expulsion of the stent. Their overall anastomotic salvage rate was 86.4%, with all of those patients achieving stoma reversal and only two requiring further surgery for a chronic fistula. They reported no problems with anorectal pain or tenesmus.

**Table 1:** The role of self-expanding metallic stents in the management of colorectal anastomotic leak

Ref	Study type	Level of defect	Cohort Size	Patient selection	Other endoscopic intervention	Faecal diversion	Other surgical intervention	Long-term salvage	Other endpoints described/ complications
Abbas [17]	Case report	Ileorectal	1	Patient declined further Sx T 38.5, HR115, WCC 12	Repeat stenting and secured with clips due to migration twice	0%	0%	100% (*n* = 1)	Stent migration 100% (*n* = 1) Stent removed day 40 No stricture at 6 months
Amrani [18]	Prospective cohort	Colorectal (*n* = 1) Ileoanal (*n* = 2)	3	Not stated 66% (*n* = 2) complete disunion	33% (*n* = 1) pigtail stent to abscess cavity 33% (*n* = 1) washout of abscess cavity	100% (66% (*n* = 2) at primary Sx) 33% (*n* = 1) after diagnosis of leak	33% (*n* = 1) drainage of abscesses	100% (*n* = 3)	Removal of stents at 5–8 weeks 33% (*n* = 1) developed stricture—required dilatation
Chi [19]	Case series	Colorectal	12	Not stated	0%	Not stated	16.70%	83.30% (*n* = 10)	Stent migration 66.7% (*n* = 8) Anorectal pain 58.3% (*n* = 7) Faecal incontinence 25% (*n* = 2) Enterocolic fistula 8.3% (*n* = 1) Mortality 0% Median time to healing 13 days
Chopra [5]	Retrospective cohort	Colorectal	6	No evidence of persisting severe sepsis leaks < 50% of the anastomosis	100% (*n* = 6) endoscopic debridement at time of stent placement	33% (*n* = 2) after diagnosis of leak	0%	100% (*n* = 6)	All leaks < 2 cm, remained in situ for median 9 days Median duration of healing 105 days Long-term intestinal continuity 77% Mortality 0%
Cooper [20]	Case series	Colorectal	3	Not stated	0%	Not stated	0%	100% (*n* = 3)	Stent migration 0% Anorectal pain—mild—oral analgesics in 66% (*n* = 2) No stenosis at 4 months Period of stent placement—mean 45 days. 15–20 cm from anal verge
DiMaio [21]	Case series	Colorectal	5	‘When surgical diversion was deemed necessary’	60% Fibrin glue (*n* = 3) +/- clip (*n* = 1) at time of stenting	20% (*n* = 1) after diagnosis of leak and failed stent	Percutaneous drain placement to abscess 80%	80% (*n* = 4)	Pain requiring admission or stent removal 60% Stent migration 40% (mean day 8) Period of stent placement—median 20 days
Lamazza [22]	Prospective cohort	Colorectal	22	Leak confirmed on gastrograffin enema	27.30% (*n* = 6) further stent after spontaneous expulsion	68% (*n* = 15) after diagnosis of leak	9% (*n* = 2) closure of rectovaginal fistula	86.4% (*n* = 19)	All leaks at leaks > 30% circumference Mean 24-month follow-up Mortality 0% Anorectal pain/tenesmus 0% Stoma closure rate 86.4%
Manta [23]	Case series	Colorectal	4	‘Patients referred to the endoscopy unit’	100% (*n* = 4) clips to close defect prior to stent	Not stated	75% (*n* = 2, operative intervention, n = 1 percutaneous drain)	50% (*n* = 2)	Mean diameter of defect 35 mm
Perez [24]	Case series	Colorectal	2	Patients declined surgery	50% (*n* = 1) VAC therapy for 2 weeks 100% clips to secure stent	50% (*n* = 1) at primary surgery	0%	100%	Stent migration 0%
									Mean time to closure − 14 weeks

Chi et al. experienced similarly promising salvage results in their case series of 12 patients, but different findings with regard to local stent symptoms [19]. Stent migration was experienced in 66.7%, anorectal pain in 58.3% and faecal incontinence in 25.0%; however, clinical success without reoperation was achieved in 83.3% of patients.

Chopra et al. [5] retrospectively compared the outcomes of 20 patients following a colorectal anastomotic leak managed with either surgical vs. endoscopic intervention. Seven underwent reoperation in the form of a surgical repair of the anastomosis or the creation of a stoma, and of the 13 patients managed with endoscopy six were stented, five had vacuum therapy and two received fibrin to close the defect. Although this was a small study, they found significant improvement in the healing time of the anastomosis in the group who were managed with endoscopy (105 days endoscopic group, 173 days operative group) and also in the proportion of patients who achieved long-term intestinal continuity (77% endoscopic group, 57% operative group). All six of their patients who were stented achieved anastomotic healing and salvage.

DiMaio et al. presented their case series in 2012 focusing on covered self-expanding metal stents in the non-operative management of post-operative colorectal anastomotic leaks [21]. They included defects less than 5 cm from the anal verge, and concurrent use of clips or fibrin glue was left to the discretion of the endoscopist. Five patients underwent the procedure, all as a result of a rectal anastomotic leak. Deployment was achieved in all, with fibrin glue used in three. Stents were removed at a median of 20 days (range 7–78), with one patient experiencing spontaneous expulsion. Complete defect resolution was achieved in two patients, with a further two patients experiencing a small residual fistula but with no requirement for further treatment. One patient was returned to theatre for formation of a defunctioning stoma due to a persistent symptomatic fistula.

A systematic review of the use of stents for colorectal anastomotic complications including was published by Arezzo et al. in 2017 [16]. Thirty-two studies were included (one multi-centre study) including 223 patients. Indications for stent placement included anastomotic leak (18 patients), fistula formation (20 patients) and luminal stricture (185 patients) in the rectum or sigmoid colon. The overall estimated early success rate was reported as 73.3%, with 9.3% of patients requiring surgical intervention. Longer term success was achieved in 57.3% of patients. The rate of stent migration was 41.5%, persisting dehiscence 25.5%, persisting stenosis 44.0% and 26.0% required secondary balloon dilatation. The authors concluded that a stent could be considered in the early post-operative management of anastomotic complications in patients who have minimal risk of sepsis, although safety and efficacy needed to be further established.

Overall although there are limited data on the use of stents for colonic and rectal anastomotic leaks, the data appear promising with most patients achieving healing of the defect. Although local symptoms of pain and tenesmus in some studies are common, this could possibly be overcome by ensuring stent placement at least 5 cm from the anal verge. Migration of the stent is a common problem throughout the studies. Although this is expected due to colonic peristalsis and the use of covered stents, it incurs extra costs when the stent is replaced and creates further interventions for the patient. Very few studies stated how they selected their patients for stenting over surgery, and the defects varied from 30 to 100% of the anastomotic circumference. In addition to this, faecal diversion varied greatly and overall was 56.4% (22/39) in the studies that reported it. It is therefore difficult to recommend which cohort this technique would be suitable for.

### Endoscopic clips

A reliable endoscopic clipping technique for anastomotic leak, iatrogenic perforations and staple line bleeding has been under development for several years. The efficacy of an initial ‘through-the-scope clip’ (TTSC) system was limited by the width of the clip branches and the limited pressure that could be applied to the tissue, often requiring multiple clips to close one small defect. Evidence was often anecdotal, and its successful translation into clinical practice limited [25]. The ‘over-the-scope clip’ (OTSC) system, first described in 2007 for the management of acute gastro-intestinal bleeds or perforations, has had much more encouraging results due to its ability to grasp larger amounts of tissue and create a higher compression force [26].

There were 7 case series and cohort studies with a total of 62 patients having clips utilised to close a defect in the colonic wall or anastomosis [23, 25–30]. A summary of the available evidence is shown in Table 2. Overall, the success rate of over-the-scope clips was reported between 57.1 and 100%, including patients who were clipped post-endoscopic iatrogenic perforation or post-operative anastomotic leaks.

**Table 2:** The role of ‘over-the scope’ endoscopic clips in the management of colorectal anastomotic leak

Ref	Study type	Level of defect	Cohort size	Patient Selection	Other endoscopic intervention	Faecal diversion	Other surgical intervention	Long-term salvage	Other endpoints described/complications
Arezzo [27]	Case series	Colorectal (*n* = 13) Ileocolic (*n* = 1)	14 (8 acute, 6 chronic)	Not stated	28.50%(Stent 7.1% (*n* = 1)) ENDOVAC 21.4% (*n* = 3)	14.3% (*n* = 2) after diagnosis of leak	7.10% (*n* = 1 patient declined clips)	86% (*n* = 12)	Complications 0% Closure confirmed on contrast enema Mean diameter of defect 9.1 mm
Kirschniak [26]	Case series	Colonic	4 (post-polypectomy perforation)	During initial endoscopy	0%	0%	0%	100%	Complications 0%
Kirschniak [28]	Case series	Colorectal	7 (4 post-scope perforations, 3 chronic fistulae)	Not stated	0%	0%	0%	57.10%	Treatment success
									100% (*n* = 4) post-polypectomy/colonoscopy perforation, 0% for chronic fistulas
Kobayashi [29]	Case series	Colorectal	2	Small leaks, failure of conservative Rx (TPN and antibiotics)	50% (*n* = 1) irrigation of abscess cavity	50% (*n* = 1) at primary surgery	0%	100%	Complications 0%
Manta [23]	Case series	Colorectal	17	‘Patients referred to the endoscopy unit’	23.50% (*n* = 4) stent at time of clip placement	Not stated	47.1% (*n* = 8) (*n* = 2 radiological drain, *n* = 6 operative intervention)	64.70%	Successful leak closure verified by endoscopic or radiological assessment Mean diameter of defect 12 mm no stent, 35 mm with stent No mortality
Mennigen [25]	Case series	Colorectal	3 (chronic leak/fistula)	Defect < 1–2 cm, viable tissue	33% (*n* = 1) endoscopic lavage	33% (*n* = 1) at primary surgery	66% (*n* = 2 laparotomy and excision of fistula)	66% (*n* = 2)	Complications 0%
Voermans [30]	Prospective cohort	Colorectal	13 (8 post-scope, 4 post-polypectomy)	Defect < 3 cm Presented within 24 h of perforation	0%	0%	7.70%	92.30%	Successful closure defined as no endoscopic and fluoroscopic evidence of a leak plus no adverse events at 30 days 7.7% (*n* = 1) mortality—dislodged clip and peritonitis

One of the primary papers reporting this technique in 2007 examined 11 patients, three with small perforations post-polyp excision and eight with acute bleeding [26]. All eight patients achieved haemostasis with the application of only one clip, and only one iatrogenic perforation went on to require further endoscopic intervention in the form of a stent. All patients avoided the morbidity associated with surgical intervention, although all defects were small at less than 2 cm.

Manta et al. have since published a prospectively collected case series of 76 endoscopically managed post-surgical leaks involving the GI tract over a 5-year period, including 24 following rectal resection or colectomy [23]. 17 cases were managed with OTSC, of which four were also stented. The mean (range) size of the defect managed solely with over-the-scope clips was 12 (5–25) mm, with those also requiring a stent having defects measuring up to 50 mm. This technique had a 64.7% success rate, defined as complete radiological and/or endoscopic resolution at follow-up, with five patients undergoing open re-intervention and one having laparoscopic suturing. In addition to this, two patients had radiological drains placed to manage local sepsis.

Mennigen et al. published the results of a case series at their tertiary referral centre in 201325. Clips were used in 14 patients with anastomotic leak, three of which were rectal. Overall success of the technique, both endoscopically and fluoroscopically, at the time of closure and long-term follow-up in all 14 patients was reported to be 79%. Kirschniak et al. published a case series in 2011 of 50 patients using over-the-scope clips [28]; 15 required intervention for colonic bleeding (one from a stapled colorectal anastomosis) and four for free colonic perforations post-polypectomy. Primary haemostasis and colonic closure were achieved in all cases with no adverse outcomes. A prospective multi-centre cohort study was published in 2012 by the CLIPPER study group examining the use of OTSC specifically for iatrogenic perforations post-endoscopic intervention [30]. In the 13 patients with colonic perforations, a 92% closure rate was reported. Despite promising results in a multi-centre setting, anastomotic complications were not included.

Arezzo et al. published a case series in 2012, collecting data over a 42-month period for 14 patients managed with OTSC for an anastomotic leak or fistula of the colon or rectum within 60 days of surgery [27]. The mean diameter of the defect was 9.1 mm (range 5–12 mm). Eight patients had acute anastomotic leaks and six had chronic leaks, two of these patients had an established colo-cutaneous fistulae and a further two had a rectovaginal fistula. Three patients with chronic anastomotic leaks also required vacuum therapy to drain the abscess cavity, and one patient had a stent placed at the time of clipping. Overall success rates of complete closure, as assessed by soluble contrast through the working channel of the scope, were 86% and 83% in acute and chronic cases, respectively, with no clip associated complications. One patient required surgical intervention, giving an endoscopic salvage rate of 92.9%. Encouraging results have also been reported in an individual case series of two patients [29].

Endoscopic clipping has an established role in haemostasis, and now over-the-scope clips appear to have a potential role in managing colonic defects. The use of these clips, however, appear to be limited to small defects of under 2 cm and were more successful in closing acute breaches, iatrogenic perforations or acute leaks rather than chronic fistulae.

### Vacuum-assisted closure

Vacuum-assisted wound closure (VAC) devices, in particular the Endo-SPONGE® (B. Braun Medical Ltd) is an open-cell, cylindrical polyurethane sponge connected to a drainage tube linked to a vacuum system exerting constant suction [31]. VAC therapy promotes healing of wounds by enhancing formation of granulation tissue, reducing oedema, increasing vascularity and decreasing bacterial colonisation [32]. Their use is well established for post-operative wound care and is being increasingly considered as a non-surgical alternative in the management of anastomotic leaks.

A total of 14 case series or cohort studies consisting of 197 patients were reviewed [5, 23, 31–42]. A summary of these are shown in Table 3. The overall rate of anastomotic salvage in patients without generalised peritonitis and deemed suitable for vacuum therapy was 88.8% (range 66.6–100%), with very few adverse outcomes reported.

**Table 3:** The role of vacuum therapy in the management of colorectal anastomotic leak

Ref	Study type	Level of defect	Cohort size	Patient selection	Other endoscopic intervention	Faecal diversion	Other surgical intervention	Long-term salvage rate	Other endpoints described/complications
Arezzo [31]	Retrospective case series	Colorectal	3	33.3% incidental finding on routine contrast enema 66.6% febrile, noted on CT	33.30% Glue and clip at 4 months to close residual defect	100% 66.6% (*n* = 2) during primary Sx, 33.3% (*n* = 1) after diagnosis of leak	33.30% (*n* = 1, proctectomy and end colostomy, day 31)	66.60%	Nil
Chopra [5]	Retrospective cohort	Colorectal	5	No evidence of persisting severe sepsis Leaks < 50% of the anastomosis Defects > 2 cm	100% had endoscopic debridement at time of stent placement	100% (*n* = 5 defunctioning ileostomy after leak diagnosed to avoid blockage of vacuum therapy)	0%	100%	All leaks < 2 cm, remained in situ for median 11 days Median duration of healing 105 days Mortality 0%
Glitsch [33]	Prospective case series	Colorectal	17	Cavities from 2 × 2 cm to 10 × 13 cm	88.20% 15 Fibrin glue for residual defect closure		5.90% (*n* = 1 Hartmann’s procedure)	94.10%	Mean duration of drainage 21.4 days Mean 5.4 sponge changes Mean 10.7 endoscopies No benefit from a diverting stoma
Keuhn [34]	Retrospective case series	Colorectal	20	‘Signs of leak without generalised peritonitis’ Abscess confirmed on CT or endoscopy	18.20% 1 diathermy for bleeding during sponge change. 3 balloon dilatation for stenosis post treatment	95% (*n* = 19, during primary surgery)	10% (2 disruptions of anastomosis for total necrotic anastomotic dehiscence)	90%	Stoma closure rate 79% Median number of sponge changes = 6 Median time of therapy 20 days
Manta [23]	Case series	Colorectal	7	‘Patients referred to the endoscopy unit’	0%	Not stated	0%	100%	Mean diameter of defect 29 mm
									Successful leak closure verified by endoscopic or radiological assessment
Mees [35]	Prospective cohort	Colorectal	5	Signs of a leak not requiring laparotomy. Confirmed on CT. Cavity > 3 cm, < 10 cm	0%	100% (*n* = 5, during primary surgery)	0%	100%	Median duration of vacuum therapy 27 days Median number of sponge changes = 7 Mild-moderate pain scores 1 stenosis requiring dilatation
Nerup [36]	Retrospective cohort	Colorectal	13	Symptoms of a leak not requiring laparotomy. Confirmed on CT. <1month since leak diagnosed	0%	100% (*n* = 13, at primary surgery)	8% (*n* = 1, end colostomy for anastomotic stenosis)	92%	Stoma closure rate 92% Median duration of vacuum therapy 18 days Median number of sponge changes = 8
Riss [37]	Prospective cohort	Colorectal	23	Extraperitoneal anastomosis	8.70% 1 fibrin glue, 1 stent	73.90% (*n* = 17 at primary surgery)	13.10% (*n* = 3, end colostomy)	86.90%	Median time for healing 21 days Median follow-up 17 months Recurrent abscess 21.7%
Srinivasamurthy [38]	Retrospective case series	Colorectal	8	Extraperitoneal low anastomosis Diagnosed on CT or contrast enema	0%	100% (*n* = 8, at primary surgery)	25% (*n* = 1, APR for persistent perianal sepsis, *n* = 1, end colostomy for intra-peritoneal sponge placement)	75%	Stoma closure rate 62.5% with ‘good or reasonable function’
Strangio [39]	Prospective case series	Colorectal *n* = 19	25	Symptoms and signs of leak, confirmed on CT	0%	52% (*n* = 13, at primary surgery)	12% (1 patient for ureteric stent, 1 patient small bowel resection for fistula, 1 abscess drainage and disruption of anastomosis)	88%	Stoma closure rate 84.6%
		Colonic *n* = 5 Ileo-rectal *n* = 1							
vBernstorff [40]	Prospective case series	Colorectal	26	Extraperitoneal anastomosis, not requiring surgical intervention	0%	69.20% (*n* = 18 during primary surgery)	11.50% (3 end colostomies for recurrent abscesses)	88.50%	Median 30.4 vs. 71.1 days to closure in patient with and without neoadjuvant chemoradiotherapy
Van Koperen [41]	Prospective case series	Colorectal	16	Symptoms and signs of leak, confirmed on CT	6.30% 1 Significant bleed at sponge change of 500 ml	100% (*n* = 8, during primary surgery, *n* = 7 after diagnosis of leak, *n* = 1 pre-existing)	18.80% (1 end colostomy for complete dehiscence, 2 proctectomies for recurrent pelvic sepsis)	81.20%	Stoma closure rate 43.8% 75% success if Endosponge™ started within 6 weeks, 38% success if started after 6 weeks of surgery 1 (6.3%)cessation of Endosponge™ due to pain
Weidenhagen [42]	Prospective case series	Colorectal	29	Signs of a leak. Confirmed on CT and endoscopy. Dehiscence 20–75% of anastomosis. Cavity 2–0 cm	31.00% 9 fibrin to close Residual defect when < 1.5 cm	86.20% (*n* = 21 during primary surgery, *n* = 4 after diagnosis of leak)	10.3% disruption of anastomosis (2 for ischaemic necrosis and complete dehiscence, 1 for failure of pre-sacral fistula to close after 6 months)	90.30% (96.6% of all who completed therapy)	Stoma reversal rate 88% Duration of vacuum therapy 34.4 ± 19.4 days Number of endoscopic sessions 11.4 ± 6.3

The first study detailing its use for this indication was published in 2008 by Weidenhagen et al., examining the results of endoscopic vacuum therapy over a 2-year period in 34 patients with an anastomotic leak following low anterior resection [42]. 29 patients were deemed eligible to continue treatment (giving informed consent, with no development of secondary complications), 21 of whom had covering stomas at the time of initial surgery and a further four requiring faecal diversion alongside VAC therapy. Two patients required return to theatre after commencement of VAC due to anastomotic necrosis. Of these 29 patients, definitive healing was achieved in 90.3% of patients including nine (31%) requiring fibrin injections to close a resulting small defect. Ambulatory management was possible in 86.2%, with minor rectal bleeding commonly reported post-sponge change due to increased vascularity in granulation tissue.

Kuehn et al. published a further case series of 41 patients in 2016, 20 of whom suffered from a colorectal anastomotic leak [34]. Median (range) therapy duration was 23 (2–109) days with a mean (range) of 7 (2–37) sponge changes per patient. The anastomotic salvage rate was 90%, with only two patients requiring return to theatre for exteriorisation of a necrotic anastomosis. Similarly, Strangio et al. published a single centre series in Milan in 2015 [39]. Of 296 patients undergoing colorectal surgery, 40 (13.4%) patients developed an anastomotic leak. Twenty-five of these leaks were managed with VAC therapy commencing after a median of 16 days post leak diagnosis, with a median of nine applications per patient over 4 weeks. Complete healing of the leak occurred in 88% of patients, with the remaining three patients developing further complications requiring surgical intervention. The only study to consider the timing of intervention was that of Van Koperen et al. who demonstrated that earlier (less than 6 weeks) use of VAC therapy resulted in greater success of salvaging the anastomosis (75% vs. 38%) [41].

Von Bernstorff et al. [40] conducted a study of 26 patients with rectal anastomotic leaks receiving endoscopic vacuum therapy and reported an overall successful closure of cavity rate of 88.5%. In those who underwent neoadjuvant chemoradiotherapy, there was a significantly longer time to leak diagnosis, (14.7 vs. 6.6 days, *p* < 0.008), longer mean duration of treatment (31.6 vs. 12.3 days, *p* < 0.001), more sponge changes (8 vs. 3, *p* < 0.035) and longer time to achieve complete closure of the leak (30.4 vs. 71.1 days, *p* < 0.01).

Of the studies that reported the prevalence of stoma creation, 82% (142/173) of patients had faecal diversion either during their primary surgery (88%) or after the diagnosis of an anastomotic leak (17%). This was an inclusion criteria in some studies due to concerns of faeces blocking the vacuum system and preventing the therapy from working [5, 34]. However, Strangio et al. 39 successfully managed 12 patients, Bernstoff et al. [40] managed eight patients, and Riss et al. [37] managed six patients with VAC therapy without proximal faecal diversion, therefore suggesting that the lack of a defunctioning stoma is not necessarily an exclusion criteria for this therapy and future studies should help clarify this.

The use of vacuum therapy to drain peri-anastomotic abscesses and aid healing of the defects is very encouraging. In common with the other techniques, it is limited to patients who are haemodynamically stable and do not have generalised peritonitis, but also to those with an extraperitoneal anastomosis. This technique appears to be safe with minimal local symptoms or complications and in some cases has been used in the outpatient setting. There is a high long-term rate of intestinal continuity of up to 92% [36] and therefore could prove to be a solution to the difficulty of percutaneous drainage in this area. An important consideration, however, is the cost of this repeated procedure as the median number of sponge changes were between 5.4 and 11.4.

### Endoscopic drainage of intra-abdominal sepsis

A pilot study published by Blot et al. in 2016 examined the feasibility of endoscopic-guided double-pigtail stents (DPS) in the management of colorectal anastomotic leaks not associated with systemic sepsis [43] The defect in the anastomotic line was initially dilated to allow maximum drainage of intra-abdominal drainage before the drain was secured. Placement was confirmed with a CT scan, and repeated at 6-week intervals until resolution of the abscess. Over a 3-year period nine patients were managed with DPS alone, five with radiological intra-abdominal drainage followed by DPS and ten with exclusively radiological drainage (RD). All patients undergoing RD alone required no further intervention, with all patients with a defunctioning stoma at the time of primary surgery successfully progressing to closure. The overall success of endoscopic management was 78.5%, and the median number of endoscopic procedures was two. One patient required concurrent expandable stent placement and two patients required progression to laparotomy for take down of the anastomosis. Of interest, of the four patients requiring adjuvant chemotherapy all were able to undergo their treatment with the DPS in situ.

### Fibrin glue

Although fibrin glue has been most extensively investigated in the use of complex perianal fistulae [44], it has also become a novel option for the management of anastomotic leak either alone or as combination therapy. A total of three studies focusing on the use of fibrin glue for the closure of anastomotic defects were reviewed including 22 patients [5, 45, 46]. A summary of available evidence is shown in Table 4. Lippert et al. reported their retrospective case series in 2011 of patients undergoing fibrin glue repair endoscopically for fistulae and anastomotic leaks [46]. Of the 47 post-operative cases examined, 14 underwent a colonic or rectal resection with an anastomosis. Success, defined as no further management interventions, was achieved for 75% in the colon, and 16.7% in the rectum. Septic complications were reported in 28.8% of the entire cohort, and 34.6% required secondary surgical intervention. No local recurrence was reported, however, follow-up was limited.

**Table 4:** The role of fibrin glue in the management of colorectal anastomotic leak

*Ref*	Study type	Level of defect	Cohort size	Patient selection	Secondary unplanned endoscopic intervention	Secondary faecal diversion	Other surgical intervention	Long-term salvage	Other endpoints described/complications
Chopra [5]	Retrospective cohort	Colorectal	2	No evidence of persisting severe sepsis ‘Small’ leak No abscess	100% (*n* = 2) had endoscopic debridement at time of stent placement	0%	0%	100%	Mortality 0%
Del Rio [45]	Retrospective case series	Colorectal	6 colorectal (13 total GI leaks)	Low volume fistula (< 100 mls/24 h)	0%	Not stated	0%	100%	Nil
Lippert [46]	Retrospective case series	Colonic Colorectal	14 colorectal (52 total GI leaks)	Use of > 1 ml glue	53.9% of all 52 patients. (Stent, clips, histoacyrl, suture)	Not stated	42.90%	50%	Death 21.1.% of all patients (3.8% associated with fistula/leak)

Weidenhagen et al. reported their series of 34 patients who had an anastomotic leak following anterior resection, primarily treating suitable patients with vacuum-assisted therapy [42]. Once the cavity was less than 0.5 × 1 cm , the use of the vacuum ceased and in nine of their patients they used fibrin glue to definitively closure the tissue defect. 96.6% of their patients achieved closure of the anastomotic defect, although it is not stated what contribution the fibrin had to this success. Del Rio et al. published a case series of 13 patients who were treated with fibrin glue, six of whom had a rectal anastomosis [45]. The leaks were detected on post-operative day 3–9, the anastomotic defects measured 2–5 mm, and each patient underwent a mean of 3.3 treatments. All patients achieved closure of the defect as confirmed by radiological examination.

### Multi-modal therapy for anastomotic bleeding

Anastomotic bleeding can present as a severe bleed in approximately 1% of patients [47], and a single significant episode in up to 6% of patients [48, 49]. With the considerations of preserving the anastomosis and achieving safe endoscopy due to active bleeding, management can be challenging. A summary of the available evidence is shown in Table 5.

**Table 5:** Multi-modal endoscopic management of anastomotic bleeding

Ref	Study type	Level of defect	Intervention	Cohort size	Long-term salvage	Surgical intervention	Secondary unplanned endoscopic intervention	Other endpoints described/complications
Besson [48]	Case series	Colorectal (lap or open left hemicolectomy)	10—None required 9—Diagnostic endo 10—OTSC 11—Injection sclerosant 7—OTSC + injection	47	89.40%	10.6% due to size of anastomotic defect	0%	–
Malik [50]	Case series	Colonic Colorectal Ileocolic	1—Diathermy and injection adrenaline 1—Injection adrenaline 1—OTSC	6	50%	50%	16.7% further endoscopy for diathermy	–
Martinez-Serrano [51]	Case series	Colorectal	7—Anastomotic washout (saline)	7	85.70%	14.30%	0%	–
Perez [52]	Case report	Colorectal	Washout and injection of adrenaline	1	100%	0%	0%	–

Besson et al. reported their case series of 729 patients undergoing either a laparoscopic or open elective left hemicolectomy over a 9-year time frame [48]. Post-operative bleeding was experienced in 6.4% of patients, of which 97.8% had a stapled anastomosis. The development of an anastomotic leak was slightly greater in this cohort (8.5% vs. 6.5%). Of the 47 patients with bleeding, 10 resolved spontaneously without need for investigation, nine underwent diagnostic endoscopy but required no intervention, 10 had clips placed, 11 had injection of a mucosal sclerosant and seven required both haemostatic techniques. No patients required further endoscopic intervention, however, five returned to theatre due to a significant anastomotic defect demonstrated at the time of diagnostic endoscopy.

Similar incidence rates are reported across the literature with equal success. Malik et al. reported a post-operative bleeding rate of 0.8%, with three patients in their case series undergoing endoscopic therapy (adrenaline injection, diathermy or clipping) achieving control and salvage of the anastomosis in all cases [50]. Martinez-Serrano et al. reported a post-operative bleed rate of 0.5% from their cohort of 1389 patients undergoing elective laparoscopic or open resection [51]. In these seven patients, endoscopic anastomotic washout with normal saline was performed and in six patients the bleeding ceased (85.7%); only one patient had to return to theatre for reconstruction of the anastomosis.

## Discussion

It is well established that anastomotic complications have a significant impact on patient morbidity and mortality, as well as potentially delaying the commencement of adjuvant chemotherapy and increasing the risk of local recurrence [53]. Timely management is also important, as prolonged sepsis and fibrosis has been associated with impaired long-term compliance and therefore function of the neo-rectum [14, 15]. It is clear from the evidence we have reviewed that endoscopic management using stents, clips, vacuum therapy or fibrin glue may negate the requirement for surgery in selected patients suffering from colorectal anastomotic leak.

The placement of covered stents provides a feasible, relatively low risk alternative to immediate surgical intervention. The subsequent progression to surgery in the acute setting has been reported to be less than 10% [16]. Their use is not suitable, however, for those patients with systemic sepsis, or for an anastomosis encroaching on the anal verge. There is a high stent migration rate and reported side effects of discomfort or tenesmus. We can draw encouraging evidence from the existing literature for OTSC, in particular the low risk associated with the procedure compared to the morbidity of re-intervention. The evidence base, however, lies predominantly in upper gastro-intestinal bleeding and small defects (< 2 cm) from iatrogenic perforations. Evidence for their use in the management of colorectal anastomotic leaks is growing. Closure of anastomotic defects following the application of endoscopic vacuum therapy has a reported success rate of up to 92% [33]. Numbers in individual studies do, however, remain small and studies are often single centre. Pre-interventional imaging and understanding of the local anatomy are integral for establishing the approach, in terms of number and sizing of sponges. Cavity size has not been reported as a contraindication with some centres using up to three sponges in cavities measuring 20 cm [42]. Concerns for this technique lie in the frequency of sponge changes (every 2–4 days) often requiring sedation or anaesthesia. The shorter healing time, avoidance of salvage surgery and the potential for up to 86% of patients to be ambulatory may well negate the additional treatment costs [41, 42]. Complications of bleeding, circumferential anastomotic breakdown and stricture requiring dilatation are uncommon but have all been reported [17, 41, 42]. The use of fibrin glue in rectal anastomotic insufficiency has been reported with varying success. Its role appears to be most suitable in patients with small defects [45], or in combination with other treatments such as vacuum therapy [42].

There are little data in any of the studies to assist the physician in selecting patients for endoscopic salvage, beyond the starting point of physiological stability. Selection of the particular endoscopic strategy is most likely to be determined by local expertise. Accepting the small evidence base for fibrin glue, this is most likely appropriate for very small defects or as an adjunct to other therapies when a small defect remains. One has to consider the possibility that defects of this size may heal with conservative treatment alone. Most of the evidence for OTSC is in the management of small iatrogenic perforations, and this technique will similarly lend itself to small defects in an otherwise healthy anastomosis. Placement of a SEMS in the colon is most commonly performed as a combined endoscopic-fluoroscopic procedure, and therefore requires availability of two operators and an interventional radiology suite. The logistical difficulties of vacuum therapy, specifically the multiple and frequent sponge changes, have already been discussed. Ultimately if a patient is felt suitable for endoscopic salvage of a deficient colorectal anastomosis, an individualised treatment plan is required. This must take into account local expertise, availability of specialist equipment, and patient factors such as the anatomy of the anastomotic defect, co-morbidity and ability to tolerate failure of therapy.

Whilst technical success and avoidance of re-operative surgery is clearly an advantageous starting point, we must consider the potential sequelae of endoscopic salvage both in terms of the risk of chronic pelvic sepsis and adverse functional outcomes. Nesbakken et al. [15] directly compared the functional outcomes of 11 patients post anastomotic leak and subsequent stoma closure with 11 patients undergoing an uncomplicated low anterior resection. Patients who had experienced leakage had a trend towards reduced neorectal capacity (*p* = 0.04), faecal urgency (*p* = 0.09) and incontinence (*p* = 0.06). Hallbook et al. [14] showed a similar picture in their study of 19 patients. Ashburn et al. [54] followed up 52 patients with an anastomotic leak following restorative colorectal resection. They identified a significantly worse SF-36 physical and mental component scale at 1 year. Kiely et al. [55] also showed reduced quality of life (*p* < 0.001) and increased daytime leakage in patients with chronic pelvic sepsis post-pouch formation. Mongin et al. [56] described poorer outcomes for lifestyle, coping/behaviour, depression and self-perception for patients with an anastomotic leak post-sphincter saving total mesorectal excision on FIQL scores. As none of these papers focused upon endoscopic anastomotic salvage, it is vital that future studies which do strive to determine the relationship between technical success and functional outcomes.

In conclusion, endoscopic therapy in the management of stable patients with colorectal anastomotic leaks appears safe and in selected patients is associated with high rates of technical success. Challenges remain in selecting the most appropriate strategy and understanding the functional and long-term sequelae of this approach. There is little evidence available detailing functional outcomes after anastomotic salvage. Large prospective cohort studies are needed to further evaluate the role of these novel strategies with a focus on patient reported outcome measures as the primary outcome rather than technical success alone.
